# Ecophysiology of *Zetaproteobacteria* Associated with Shallow Hydrothermal Iron-Oxyhydroxide Deposits in Nagahama Bay of Satsuma Iwo-Jima, Japan

**DOI:** 10.3389/fmicb.2015.01554

**Published:** 2016-01-11

**Authors:** Tatsuhiko Hoshino, Takashi Kuratomi, Yuki Morono, Tomoyuki Hori, Hisashi Oiwane, Shoichi Kiyokawa, Fumio Inagaki

**Affiliations:** ^1^Japan Agency for Marine-Earth Science Technology, Kochi Institute for Core Sample ResearchNankoku, Japan; ^2^Japan Agency for Marine-Earth Science Technology, Research and Development Center for Submarine ResourcesNankoku, Japan; ^3^Department Earth and Planetary Sciences, Kyushu UniversityFukuoka, Japan; ^4^Environmental Management Research Institute, National Institute of Advanced Industrial Science and TechnologyTsukuba, Japan; ^5^Mishimamura VillageKagoshima, Japan

**Keywords:** iron-oxidizing bacteria, *zetaproteobacteria*, shallow hydrothermal vents, iron cycle, network analysis, fluorescence *in situ* hybridization (FISH), MiSeq Illumina, polyphosphates granules

## Abstract

Previous studies of microbial communities in deep-sea hydrothermal ferric deposits have demonstrated that members of *Zetaproteobacteria* play significant ecological roles in biogeochemical iron-cycling. However, the ecophysiological characteristics and interaction between other microbial members in the habitat still remain largely unknown. In this study, we investigated microbial communities in a core sample obtained from shallow hydrothermal iron-oxyhydroxide deposits at Nagahama Bay of Satsuma Iwo-Jima, Japan. Scanning electron microscopic observation showed numerous helical stalk structures, suggesting the occurrence of iron-oxidizing bacteria. Analysis of 16S rRNA gene sequences indicated the co-occurrence of iron-oxidizing *Zetaproteobacteria* and iron-reducing bacteria such as the genera *Deferrisoma* and *Desulfobulbus* with strong correlations on the sequence abundance. CARD-FISH indicated that the numbers of *Zetaproteobacteria* were not always consistent to the frequency of stalk structures. In the stalk-abundant layers with relatively small numbers of *Zetaproteobacteria* cells, accumulation of polyphosphate was observed inside *Zetaproteobacteria* cells, whereas no polyphosphate grains were observed in the topmost layers with fewer stalks and abundant *Zetaproteobacteria* cells. These results suggest that *Zetaproteobacteria* store intracellular polyphosphates during active iron oxidation that contributes to the mineralogical growth and biogeochemical iron cycling.

## Introduction

Members of the class *Zetaproteobacteria* were first found at the Loihi Seamount, Hawaii (Moyer et al., 1995). *Mariprofundus ferrooxydans* is the only isolate characterized as a marine iron-oxidizing bacterium within the *Zetaproteobacteria* (Emerson et al., 2007). Previous studies of 16S rRNA genes in deep-sea hydrothermal fields showed that members of the *Zetaproteobacteria* are widely distributed in iron-oxyhydroxide deposits on the plate spreading centers, hot-spot seamounts, and island arcs (Davis et al., 2009; Kato et al., 2009; Forget et al., 2010; Edwards et al., 2011; McAllister et al., 2011; Fleming et al., 2013). These observations strongly suggest that microbial communities involving *Zetaproteobacteria* play significant ecological roles in biogeochemical iron and other elemental cycles.

In the deep-sea hydrothermal iron-oxyhydroxide deposits, it has been demonstrated that the dissolved oxygen is present but generally lower than that of the surface seawater, e.g., less than 50 μM of oxygen was observed in iron-rich mats around the Loihi Seamount (Glazer and Rouxel, 2009), suggesting that members of the *Zetaproteobacteria* preferentially inhabit and grow by oxidizing ferrous iron to ferric iron at the sub-oxic redox condition. Consistently, a kinetic model study using a pure culture supported the notion that the habitable zone of iron-oxidizing microorganisms is severely and sensitively constrained by *in situ* oxygen concentration, and the maximum value for the geochemical niche approaches ~50 μM (Druschel et al., 2008). In addition, a genomic study of *Mariprofundus ferroxydans* PV-1 (Singer et al., 2011), which was isolated from hydrothermal venting at Loihi Seamount, revealed that it has the complete TCA cycle, the ability to fix CO_2_, and genes encoding aerotaxis as well as antioxidant functionalities. Although strain PV-1 does not always represent metabolic pathways and functions of all iron-oxidizing *Zetaproteobacteria*, the genomic information suggest that this strain is capable of sensing and responding to the redox state of the iron-oxyhydroxide deposits. Comparative genomic analyses of single-amplified genomes also indicated the niche specialization of *Zetaproteobacteria*, most likely controlled by the oxygen tolerance (Field et al., 2015). However, ecophysiology and growth/survival strategy of *Zetaproteobacteria* correlated with other members in the iron-oxidizing microbial ecosystem are still largely unknown.

**Figure 1:**
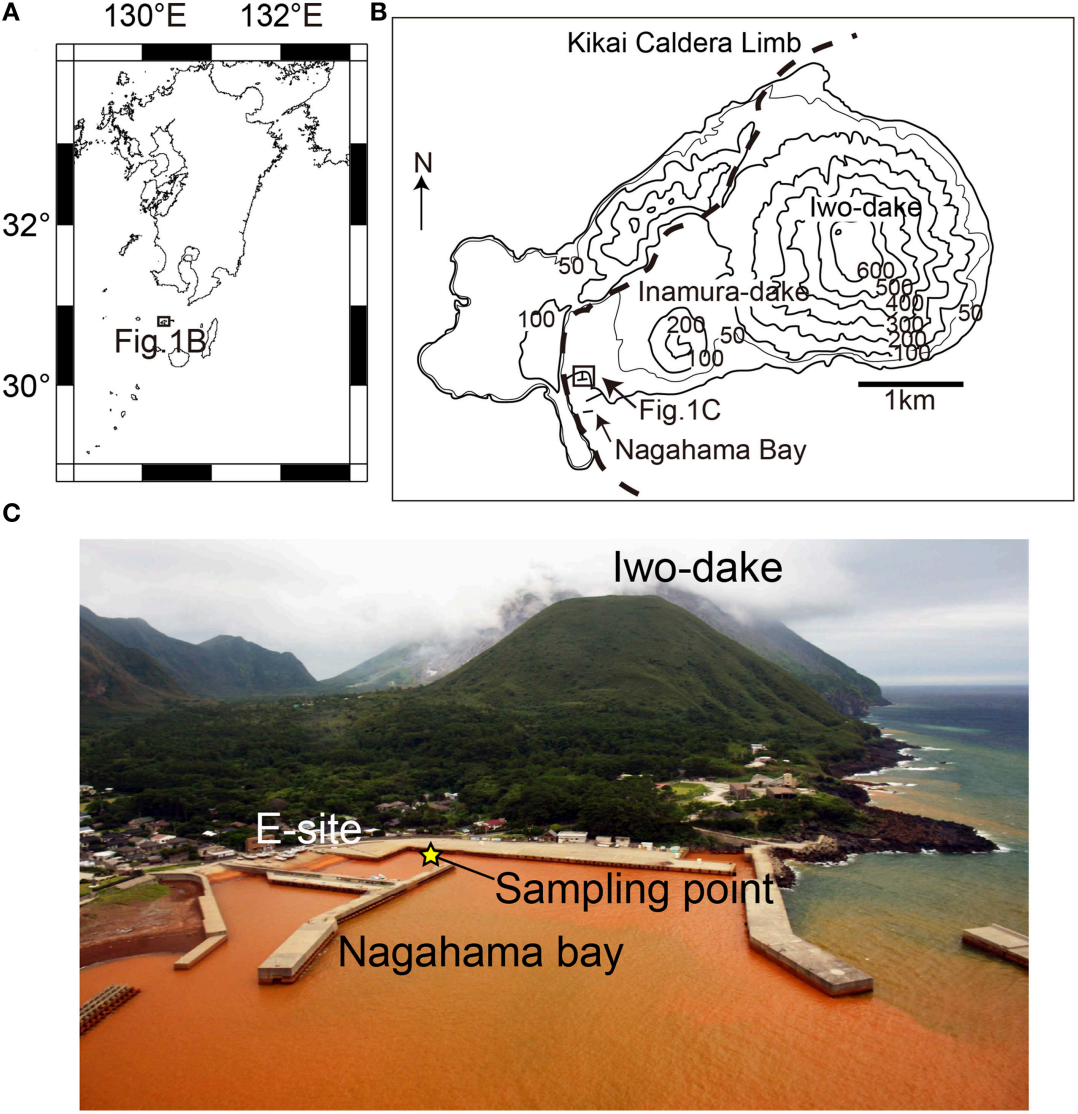
**Regional (A) and local (B) maps of Satsuma Iwo-Jima**. **(C)** An overview photo of the Nagahama Bay. The seawater is brownish-red due to the presence of iron oxyhydroxides. The yellow star indicates the sampling point in this study.

Satsuma Iwo-Jima is a small volcanic island located at ~40 km south of Kyushu Island, Japan. The volcanic activity provides a shallow hydrothermal field in the Nagahama Bay, where the formation of iron-oxyhydroxide deposits, including chimney-like structures was widely observed on the seafloor (Figure 1). Pilot geological and sedimentological studies of this environment showed that the depositional rates are exceptionally high, ranging from 2.8 to 4.9 cm per year (Kiyokawa and Ueshiba, 2015). Light microscopic observation of these deposits showed twisted stalk structures, suggesting the occurrence of iron-oxidizing microbial communities that mediate the formation process of iron-oxyhydroxide deposits.

In this study, we investigated microbial communities in the shallow hydrothermal iron-oxyhydroxide deposits (water depth: ~3 m) in the Nagahama Bay. To understand the distribution and ecophysiological characteristics of *Zetaproteobacteria* cells in this iron-rich habitat, we obtained a 50 cm-long core sample and studied microbial communities using scanning electron microscopy (SEM), image-based cell count, and catalyzed reporter deposition-fluorescence *in situ* hybridization (CARD-FISH) techniques as well as diversity and correlation analyses of 16S rRNA gene-tagged sequences.

## Materials and methods

### Geologic setting

Satsuma Iwo-Jima is a small volcanic island of the southern Kyushu, Japan. The volcanism is associated with iron-rich sedimentations at hydrothermal hot springs along the island coast. The Nagahama Bay is located on the southwest coast of the island and is one of the most active regions of hot water discharge (Figure 1). The seawater is reddish-brown because of the oxidation of ferrous iron in hydrothermal fluids as it mixes with seawater (Nogami et al., 1993; Kiyokawa et al., 2012). The hot waters discharged along the coast of Nagahama Bay (55–60°C and pH 5.5) is microaerobic (Eh = 69 mV) and contain high concentrations of ferrous iron (~191 ppm). The bottom seawater around iron deposits in the bay is anaerobic to micro-aerobic (Shikaura and Tazaki, 2001).

### Sample collection

**Figure 2:**
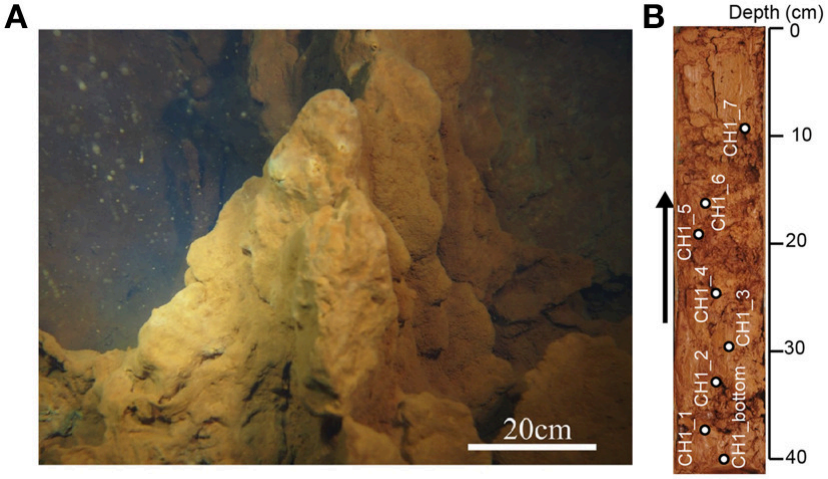
**(A)** The shallow hydrothermal iron-oxyhydroxide deposits off Satsuma Iwo-Jima. **(B)** A 50 cm-long core sample examined in this study. Arrow indicates the direction of the core, with the right side representing the seafloor. White circles indicate sampling points.

A 50-cm-long sediment core was collected from an iron-rich hydrothermal mound in the Nagahama Bay in May 2013 by scuba divers using a clear acrylic tube (Figure 2). For DNA and SEM analyses, 10 cm^3^ subsamples were collected from the split core and stored at −20°C until further processing. There are hard and soft layers inside the core and the subsamples were only taken from relatively soft and undisturbed layers in this study (Figure 2B). For microscopic analyses such as cell count and fluorescence *in situ* hybridization (FISH), 10 cm^3^ of sediments were fixed with 4% paraformaldehyde, washed twice with 1 × PBS, re-suspended in 50% PBS-ethanol solution, and then stored at −20°C.

### Scanning electron microscopy

Sample preparation and observation by a SEM was conducted as previously described (Kiyokawa and Ueshiba, 2015). The frozen iron-oxyhydroxide sample was dried in a freeze dryer (IWAKI, FRD-82M), and then subsequently fixed on a microscope slide using conductive tapes. The slide was coated with a 30-nm platinum film using an ion-sputtering device (JEOL, JFC-1600). The coated samples were analyzed by a field emission-SEM (JEOL, JSM-6500F).

### Cell count of SYBR green i-stained cells

Cell concentration in sediment samples was determined based on a previously described procedure (Morono et al., 2013). Briefly, after washing fixed sediment using NaCl solution, a detergent mix and methanol were added and the sediment was shaken for 60 min at 500 rpm using a Shake Master (Bio Medical Science, Japan). Then, the sediment slurry was sonicated and carefully layered onto a high-density cushion consisting of multiple Nycodenz and sodium polytungstate solutions. Following centrifugation at 10,000 × g for 60 min, the supernatant was transferred to a separate vial. Subsequently, detergent mix and methanol were added to the remaining pellet, followed by sonication and centrifugation with the high-density cushion solution. The resulting supernatants were pooled, and 10% of the recovered suspension was filtered on a polycarbonate membrane. The number of cells stained by SYBR Green I was enumerated using an automated cell count system (Morono and Inagaki, 2010), with analysis of acquired images using MetaMorph software (Molecular Devices, USA).

### CARD-FISH and DAPI-stain of *Zetaproteobacteria* cells

CARD-FISH was employed to detect and quantify *Zetaproteobacteria* in this study. A new probe, Zeta709 (5′-GCCTCAGGTGTTCCTCCG-3′), was designed in this study for the specific detection of *Zetaproteobacteria*. The optimal formamide (FA) concentration in the hybridization buffer was determined by clone-FISH (Schramm et al., 2002). Briefly, using ARB software (Ludwig et al., 2004), the specific probe was designed for targeting all *Zetaproteobacteria* in the public databases, including nearly full-length of *Zetaproteobacteria* sequences obtained in this study. To find the optimal FA concentration for CARD-FISH, the clones were tested at diffrent FA concentrations from 0 to 80%, resulting in 60%. Then, hybridization was carried out using microbial cells placed on a polycarbonate filter with 60% FA at 46°C for 2 h, followed by tyramide signal amplification using Alexa555-labeled tyramide at 37°C in the dark for 30 min (Hoshino et al., 2008). DNA was then counterstained using SYBR Green I. Polyphosphate inside cells was stained using a high concentration of DAPI solution (80 μg ml^−1^), if needed (Serafim et al., 2002).

### DNA extraction and PCR amplification

DNA was extracted from 2 g of frozen sediment using a PowerMax® Soil DNA isolation kit (MO BIO Laboratories, USA) according to the manufacturer's instructions. The extracted DNA was further concentrated by isopropanol precipitation. For clone-FISH, nearly full-length 16S rRNA was amplified using a 27F-1492R primer set (Lane, 1991; Loy et al., 2002), whereas a 27F-927R primer set (Amann et al., 1995) was used for fine classification of *Zetaproteobacteria*. After purification, the amplicon was cloned, and then the sequence was determined by Sanger sequencing as described elsewhere. For analyzing community structure, the V4 hyper-variable region of 16S rRNA was amplified using the universal primer set 515F-806R (Lane, 1991; Caporaso et al., 2011). After indexing using the barcoded primers, illumina sequencing was performed using a MiSeq platform with MiSeq Reagent Kit v2 500 cycles (Illumina, USA).

### Processing of sequence data and statistical analysis

Sequence data were processed using the Mothur software package (v.1.35.0) (Schloss et al., 2009). Spearman's rank correlation coefficients were calculated using the phylogenetic compositions by Mothur, and the data were illustrated using Cytoscape (v.3.2.0) (Shannon et al., 2003). For the data illustration, we used only statistically significant correlations (*p* < 0.05) with coefficients greater than 0.9. A phylogenetic tree was constructed using MEGA5 software (Tamura et al., 2011). ARB software was used for designing an oligonucleotide probe specific for *Zetaproteobacteria*.

### DNA sequences in the databases

The sequence data have been submitted to the DDBJ/EMBL/GenBank databases under accession number LC074716 to LC074722 and DRA004034.

## Results

### Cell concentrations and abundance of stalk structures

**Figure 3:**
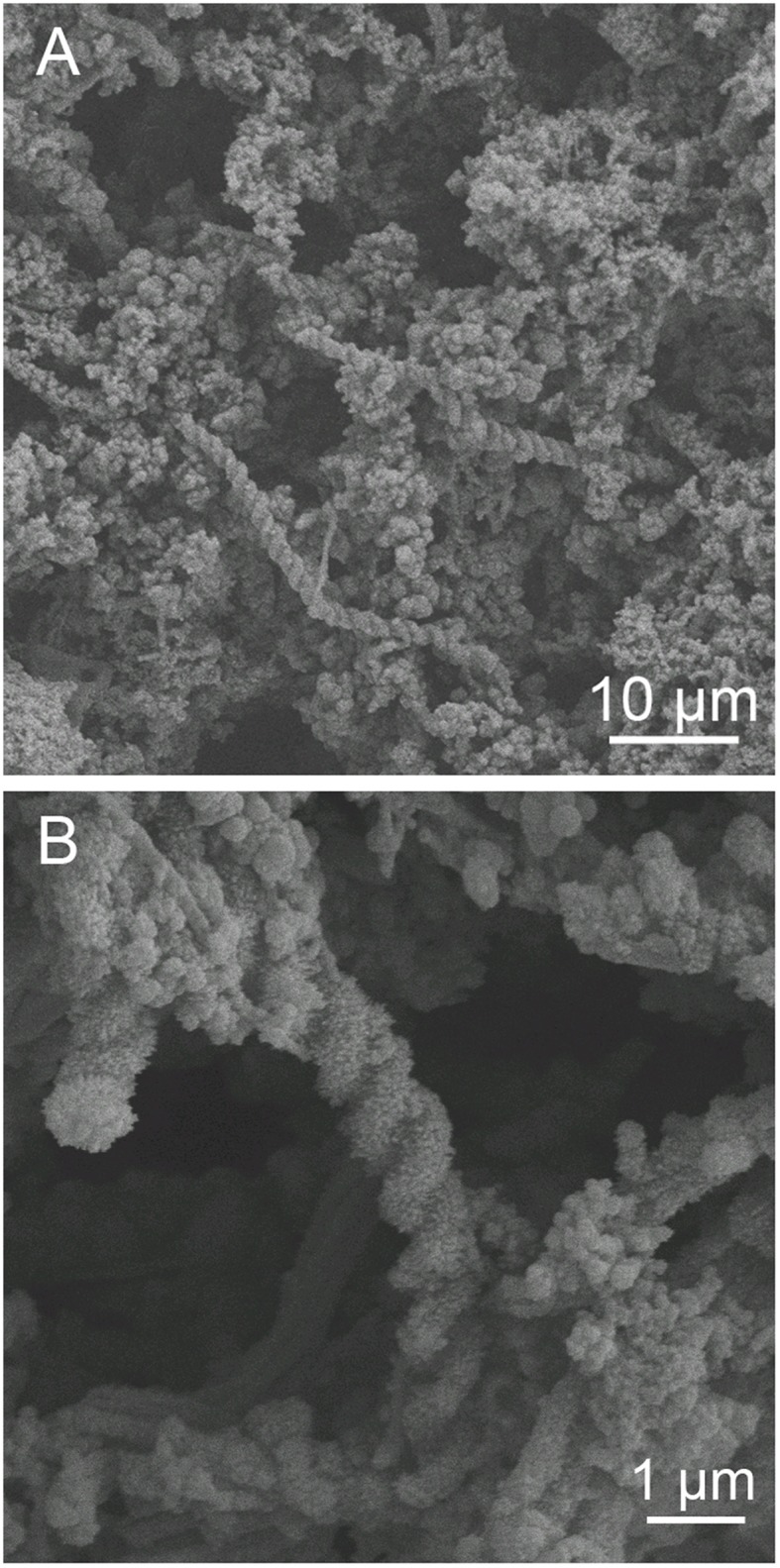
**Scanning electron micrograph of iron oxyhydroxides**. **(A)** Overview of iron-oxyhydroxide morphology. Helical stalk-like structures and particulate deposits on the surface were observed. **(B)** A higher-magnification image of **(A)**. Fine particulate minerals adhere to the stalks. Behind the stalks, ribbon-like structures were observed.

**Figure 4:**
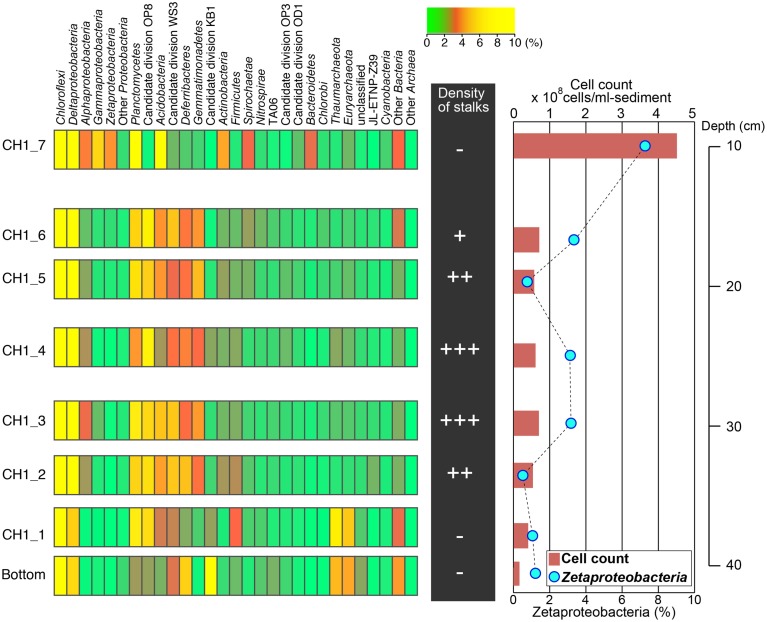
**Left:** Microbial community structures based on phylum-level classification using 16S rRNA gene-tagged sequences. Phyla comprising higher than 10% of the total sequence reads are shown in yellow. **Middle:** Relative abundance of stalks observed by light microscopy. **Right:** Cell concentrations estimated by SYBR Green I staining (red bars) and the relative abundance of *Zetaproteobacteria* detected by CARD-FISH (blue circles; also see Figure 5).

The abundance of cells and stalk structure was determined in different depths (10–40 cm from the seafloor) of the 50 cm-long core dominated by red-brownish iron deposits (Figure 2). SEM observations showed the twisted stalk structures, which are morphologically similar to those previously reported as biogenic iron oxyhydroxides (Krepski et al., 2013; Figure 3). However, other morphotypes such as sheath-cased structures (Fleming et al., 2013) were not observed in this environment. Fine particulate precipitates were observed on the surface of the stalks, suggesting that the structures function as nuclei for the growth of iron minerals. Abundance of those helical stalk structures was also evaluated by using a light microscopy. In the topmost and bottom layers (CH1_7, CH1_1, and CH1_bottom), only a few stalk structures were observed, whereas the abundance increased in the middle layer sediments (CH1_2 to CH1_6, Figure 4, middle).

Fluorescent image-based cell count showed that the number of microbial cells was highest in the topmost CH1_7 at 4.23 × 10^8^ cells cm^−3^ and declined sharply in CH1_6 to 7.26 × 10^7^ cells cm^−3^ (Figure 4, right). Cell concentrations in iron-oxyhydroxide deposits below CH1_6 were relatively constant at around 6 to 7 × 10^7^ cell cm^−3^ down to the sample CH1_2, and then decreased again to 1.65 × 10^7^ cells cm^−3^ in the deepest sample CH1_bottom.

### Taxonomic composition and microbial community structure

Illumina sequencing provided around 100,000 16 S rRNA sequences per sample after removing chimeras and low-quality sequences. Taxonomic analysis of the 16S rRNA sequences indicated that members within the phylum *Chloroflexi* are the most predominant microbial component throughout the 50 cm-core column, representing 35–40% of the total sequence reads in each sample (Figure 4). Sequences related to *Deltaproteobacteria*, including sulfate-reducers, were also abundant, accounting for 5–15% of the total sequence reads in all depth horizons. Sequences related to *Zetaproteobacteria* were prominent only in the topmost layer, composing 4% of the total sequence reads. The proportion of *Zetaproteobacteria* sequences decreased down to ~1% or less in the middle and bottom layers.

In the middle layers from CH1_2 to CH1_6 where the stalk structures were frequently observed, *Deferribacteres* represented 3–4% of the total sequence reads. Almost all of the *Deferribacteres* sequences were closely related to the genus *Caldithrix*, which are known as thermophilic nitrate-reducing bacteria (Miroshnichenko et al., 2003). *Gemmatimonadetes* and the candidate division OP8 were also prevalent only in the middle layer, representing 3–5% and 7–9% of the total sequence reads, respectively. In the lowest layer, members of the candidate division KB1 represent 11% of the total sequence reads, but its metabolic and physiological characteristics are not predictable because of no representative isolates in this group.

As for the archaeal 16S rRNA gene sequences, members within the *Euryarchaeota* and *Thaumarchaeota* were abundant only in the bottom layer. Sequences related to the *Crenarchaeota* were barely detected in our samples. Based on the sequence frequency of archaeal 16S rRNA genes, archaeal communities are relatively minor in this bacteria-dominated sedimentary habit. Nevertheless, the archaeal sequences we detected were similar to those found in an iron-rich bacterial mat at Pele's Vents on the summit of Loihi Seamount (Moyer et al., 1998).

### Microscopic observation and quantification of *Zetaproteobacteria*

**Figure 5:**
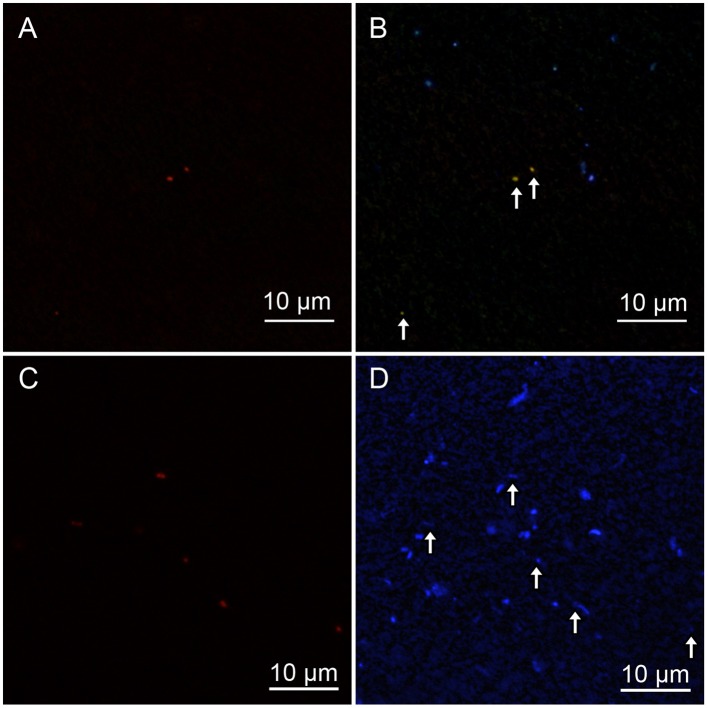
**(A,C)** *Zetaproteobacteria* detected by CARD-FISH using a Zeta709 probe. **(B,D)** High-concentration DAPI staining showed polyphosphate that emits yellowish fluorescence; blue indicates DNA inside cells. White arrows indicate *Zetaproteobacteria* cells detected by CARD-FISH. The contrast was enhanced in **(D)**, because DAPI fluorescence was faint. **(A,B)** show CH1_5 sediment, whereas **(C,D)** show CH1_7 sediment.

To determine the optimal formamide (FA) concentration for specific detection of *Zetaproteobacteria*, clone-FISH was performed using the nearly full-length of the 16 rRNA genes amplified from DNA extracted from the iron-oxyhydroxide samples. The optimal FA concentration was determined to be 60% when hybridization was carried out at 46°C. Analysis of CARD-FISH images for naturally occurring communities showed that all of the *Zetaproteobacteria* cells detected by CARD-FISH were small coccoids with the diameter of ~0.5 μm (Figures 5A,C), and they accounted for 7% of the total number of cells in the topmost layer. In the middle layer (CH1_2-CH1_6), the abundance of *Zetaproteobacteria* cells ranged only from ~1 to 3%. No clear correlations between the *Zetaproteobacteria* abundance and the frequency of helical stalk structures were observed (Figure 4, right). In the bottom layer of CH1_bottom and CH1_1, the *Zetaproteobacteria* abundance was as low as 1%, where only a few stalk-structures were observed.

Since the reported genome of *M. ferroxydans* PV-1 has genes related to polyphosphate metabolism (Singer et al., 2011), we tried to stain cells with a high concentration of DAPI, revealing that *Zetaproteobacteria* accumulate polyphosphate grains within the cells at the middle layer (Figure 5B). Interestingly, however, no polyphosphate grains were observed in *Zetaproteobacteria* cells at the top and bottom layers.

### Beta diversity of microbial communities

**Figure 6:**
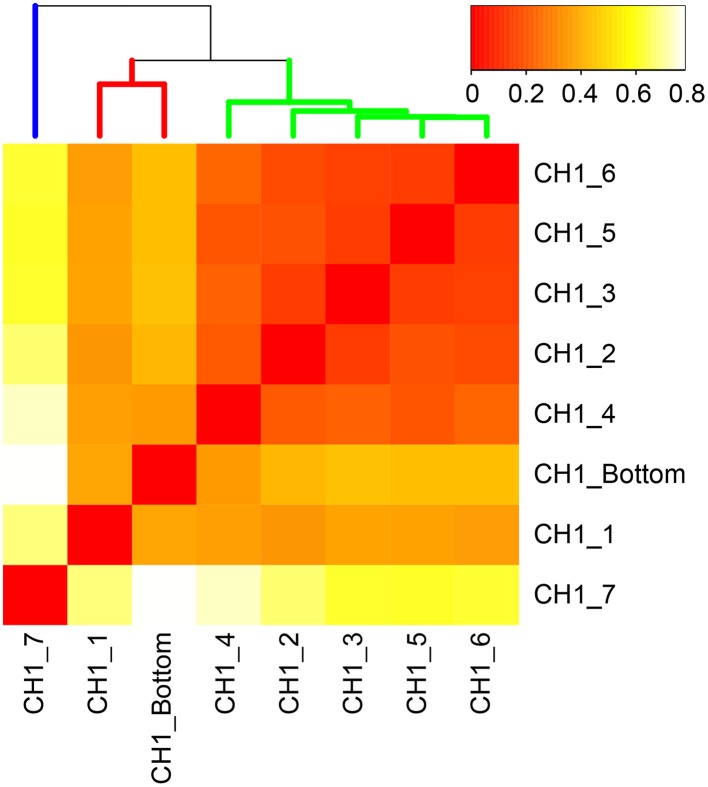
**Bray-Curtis dissimilarity matrix of microbial communities based on 16S rRNA gene-tagged sequences at the genus-level classification**. More red color indicates higher similarity. Three distinct clusters correspond to the top, middle, and bottom layers of the examined 50 cm-long core sample colored by blue, yellow, and red, respectively.

Beta-diversity of microbial communities in the iron-oxyhydroxide deposits was examined by Bray-Curtis dissimilarity analysis at the genus-level taxonomic classification (Figure 6). The result showed that microbial communities could be clustered into 3 groups corresponding to the sample depth, i.e., top, middle, and bottom. The top group (CH1_7) was markedly distinct from the other groups. The other 7 samples were more similar to each other and were categorized into two groups: the middle group (containing 4 samples) and the bottom group (containing 2 samples). In the middle group, CH_4 was relatively distinct from the other 4 samples. This zonation pattern is consistent with the occurrence of stalk structures (Figure 4, right), suggesting that there are at least 3 types of ecological niches in the cored 50 cm-long iron-oxyhydroxyide deposit.

### Phylogenetic analysis of the *Zetaproteobacteria* sequences

**Figure 7:**
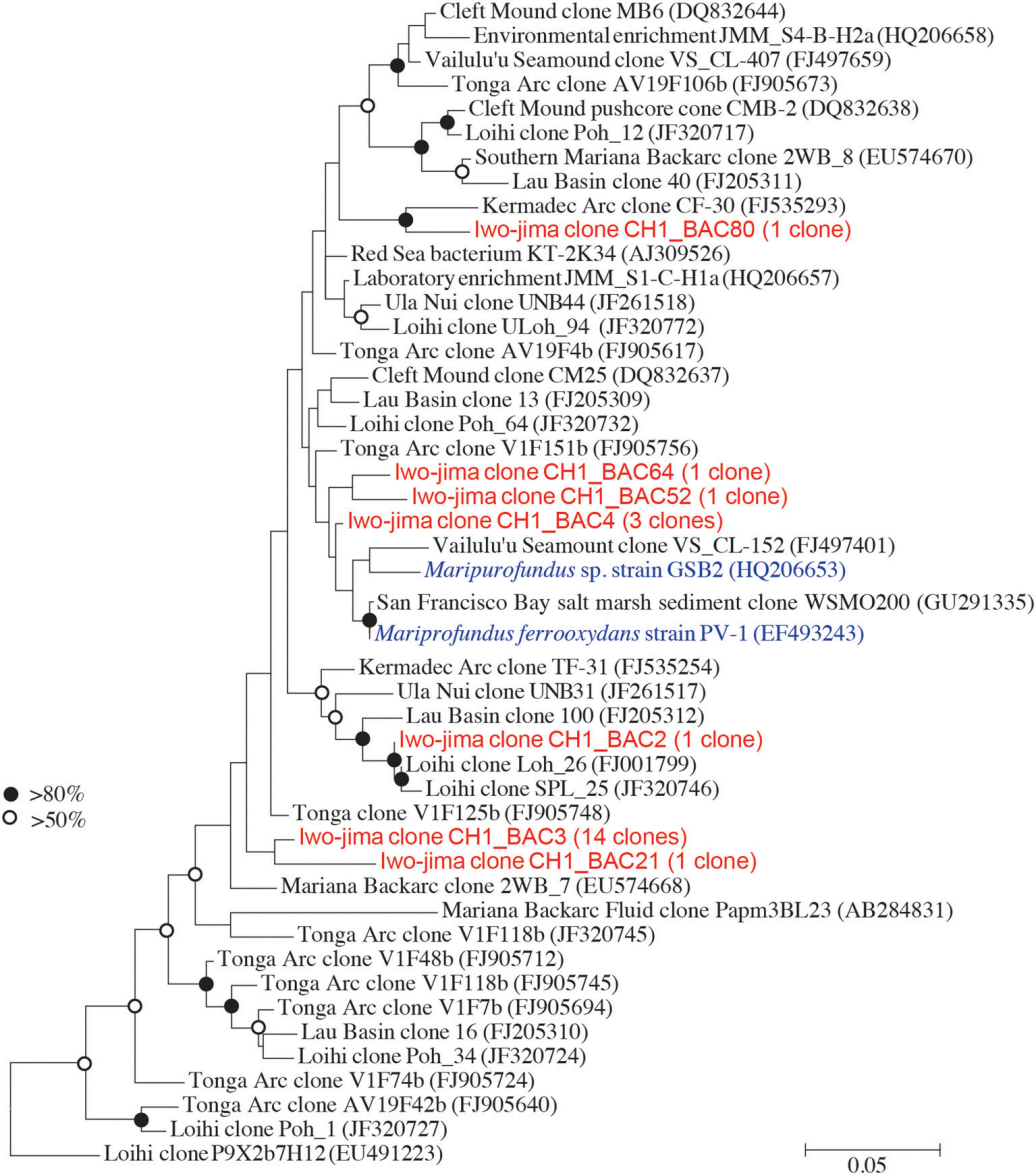
**Molecular phylogenetic analysis of ***Zetaproteobacteria*** using the maximum-likelihood method with the Jukes-Cantor model**. The V1-V4 region of 16 S rRNA sequences was used. Sequences marked in red indicate *Zetaproteobacteria* obtained in this study and in blue indicate pure cultures.

Because the length of the sequence obtained by Illumina MiSeq (254 bp) did not provide enough information for the genus-level classification, we additionally performed Sanger sequencing and obtained near-full-length sequences for the V1–V4 regions of the 16S rRNA gene, providing the finer and more precise taxonomic positions of the *Zetaproteobacteria* sequences from the examined environment. A total of 22 *Zetaproteobacteria* sequences were obtained from 331 bacteria clones in this study, although the proportions of *Zetaproteobacteria* in the Miseq sequence libraries were relatively low at around 1–5%. A phylogenetic tree was constructed based on the previously reported sequences (McAllister et al., 2011). The clone CH1_BAC3 composed 66% of the total *Zetaproteobacteria* sequences and formed a unique cluster with CH1_BAC21 (Figure 7). CH1_BAC80 and CH1_BAC2 were closely related to the sequence detected from the Kermadec Arc (Hodges and Olson, 2009) and the Loihi Seamount (McAllister et al., 2011), respectively. Other sequences such as CH1_BAC4 were closely related to sequences of *Mariprofundus ferrooxydans* and its relatives.

### Correlation between *Zetaproteobacteria* and other microbial members

**Figure 8:**
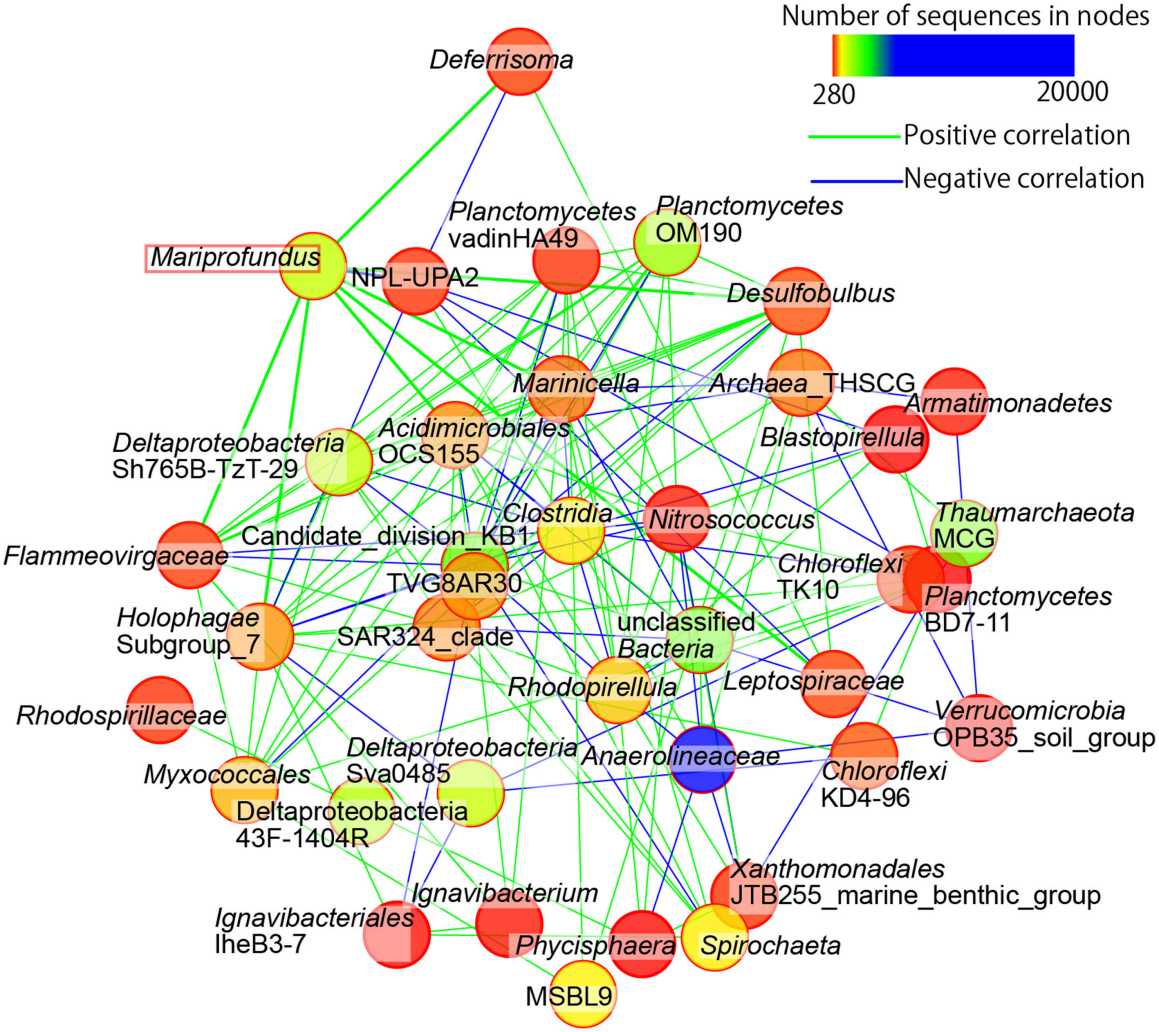
**Community networks based on Spearman's correlation analyses**. Only nodes representing first and second neighbors of *Zetaproteobacteria* (i.e., *Mariprofundus* sp.) and only strong correlations (rho values greater than 0.9) are shown. Green and blue lines indicate positive and negative correlations, respectively. Lines connected to *Mariprofundus* are drawn in bold.

We performed the community network analysis based on Spearman's rank correlation coefficient data at the genus-level classification of the sequences. Interestingly, the frequencies of iron-oxidizing *Zetaproteobacteria* strongly correlated with anaerobic or microaerophilic bacteria, such as the members of iron-reducing *Deferisoma* and sulfate-reducing *Desulfobulbus* (Figure 8). The members of *Acidimicrobiales*, which contains iron-oxidizing bacteria that can also reduce iron under reductive conditions, were also positively correlated with *Zetaproteobacteria*. The only exception was the members of *Marinicella*, which are aerobic heterotrophs positively correlated with the other microbial members. Negative correlation was observed with the candidate division NPL-UPA2, which sequences have been widely detected in both marine and terrestrial environments and their metabolic and physiological characteristics are still unknown.

## Discussion

In the 50 cm-long core sample obtained from shallow hydrothermal iron deposits in the Nagahama Bay, members of the phylum *Chloroflexi* are the most predominant microbes throughout the core column. All the *Chloroflexi* sequences were most likely non-phototrophic, mesophilic to moderatory thermophilic anaerobes and no sequences related to phototrophic members are present, e.g., members of the class *Anaerolineae* are known to be strictly anaerobic heterotrophs that have been often detected from various organic-rich sedimentary environments, e.g., terrestrial soils, hydrothermal sediments, and marine subsurface sediments (Inagaki et al., 2006; Yamada et al., 2006). In the iron deposits that we examined, these *Chloroflexi* sequences present 8–20% of the total reads in all samples, indicating that, like other sedimentary microbial ecosystems, these members may play ecological roles in biogeochemical carbon cycling. It may also be possible that *Anaerolineae* ferment organic matter and produce fermented secondary products for iron-reducing bacteria (also iron-oxidizing members, if these are mixotrophs). In addition, the phylum *Chloroflexi* has been reported to be dominant in iron reducing enrichment culture (Hori et al., 2015) and *Ardenticatena maritima* that can reduce ferric iron was isolated from iron-rich coastal hydrothermal field (Kawaichi et al., 2013). Therefore, we cannot exclude the possibility that some of the *Chloroflexi* detected here might also associate with ferric iron reduction. In fact, we observed potential sulfate reducers such as members of the *Deltaproteobacteria* throughout the cored iron deposit we examined. If some of those members have multiple metabolic functions that can utilize either iron or sulfate as an electron acceptor, the presence of high concentration of iron-oxyhydroxides might be advantageous for iron reduction (Chapelle and Lovley, 1992) and hence they might contribute to iron cycling with *Zetaproteobacteria*.

It is not surprising in this iron-rich shallow sedimentary microbial ecosystem that many 16S rRNA sequences in all the examined depths are closely related to known sulfate-reducing bacteria (i.e., *Deltaproteobacteria*). Because the prevalence of sulfate-reducing conditions down to at least 40 cm below the seafloor and hence, even through the iron deposits are soft and highly permeable for discharging hydrothermal fluids and/or the overlying seawater, the redox state within the iron deposits are heterologous but generally sub-oxic to anaerobic conditions, providing habitable zones for both *Chloroflexi* and sulfate reducers with iron oxidizers. Beta-diversity analysis of microbial communities indicated the zonation pattern of three ecological niches in the iron deposits: the top (10 cm), middle (17–33cm), and bottom layers (~40 cm), suggesting the occurrence of redox-sensitive microbial habitats associated with iron and other elemental cycles. In fact, microscopic observations revealed that twisted stalk structures are most abundant in the middle layer. In addition, based on the visual observation, upward hydrothermal fluid flows seem to form complicated webs of the fluid passage in the middle zone (Kuratomi et al., 2015), which may create the patchily heterologous redox states that biogeochemical iron and other elemental cycles preferentially occur.

The twisted stalk structure is a typical morphological feature formed by iron-oxidizing *Zetaproteobacteria* under relatively low oxygen concentrations ranged from 3 to 23 μM (Krepski et al., 2013). Because we did not find any other known iron-oxidizers such as *Marinobacter* and *Hyphomonas* in the shallow hydrothermal iron deposits that we examined, the detected members of *Zetaproteobacteria* should play a primary role in the formation process of thick iron-oxyhydroxide deposits in the Nagahama Bay. Our previous observations indicated that the vertical formation rate of the iron deposit mound is ~12 mm y^−1^ (Kuratomi et al., 2015), which is similar to the formation rate of ~19 mm y^−1^ estimated by using a pure culture of *M. ferroxydans* (Chan et al., 2011). It is also conceivable that abiotic autocatalysis on the iron-oxyhydroxide stalks may accelerate the iron deposition (Rentz et al., 2007). On the other hand, we did not observed any stalk- or sheath-like structures in the topmost and bottom layers, implying that abiotic iron oxidation is dominant or other biotic oxidation process occurs without formation of the unique structures.

The community correlation network analysis based on Spearman's rank of SSU rRNA sequences at genus-level classification also exhibited the co-occurrence of iron-oxidizing *Zetaproteobacteria* and other anaerobic respirers in the iron-oxyhydroxide deposits. For example, *Deferrisoma* and *Desulfobulbus* showed a strong correlation to *Zetaproteobacteria* (Figure 8). *Deferrisoma* is mesophilic heterotrophs that can grow optimally at 50°C and use ferric iron or elemental sulfur as electron acceptors (Slobodkina et al., 2012), and *Desulfobulbus* is characterized as sulfate and iron reducers (Holmes et al., 2004). It has been reported that some sulfate-reducing bacteria are tolerant to relatively high concentrations of oxygen and have aerobic respiration systems (Muyzer and Stams, 2008). Although it still remains unknown if these members primarily utilize ferric iron for the anaerobic energy respiration instead of sulfur compounds or even both flexible to the *in situ* redox state, the strong correlations between iron oxidizers and reducers indicate the occurrence of iron cycling in this environment.

Another interesting finding in this study would be a discrepancy between the frequency of twisted stalk structures and the number of *Zetaproteobacteria* cells. Both deep sequencing analysis of 16S rRNA and CARD-FISH consistently showed that *Zetaproteobacteria* were most abundant in the top layer (i.e., CH1_7), although fewer stalk structures were observed (Figure 4). Although the taxonomic compositions of *Zetaproteobacteria* are varied in sediment depths, OTU1 was found to dominate all sedimentary horizons where we observed stalk-like structures (see Supplementary Figure 1). We might not be able to eliminate a possibility that minor *Zetaproteobacteria* or other iron-oxidizers may contribute to iron oxidation and mineral growth at top and bottom layers, however, we infer that OTU1-relatives play a significant ecological role in this habitat. In marked contrast, the occurrence of polyphosphate-accumulated cells was observed only in the middle layer, whereas no polyphosphate accumulations were observed in the top and bottom layers. It has been reported that a diverse array of microbes are capable of polyphosphate accumulation in natural and artificial environments (Hupfer et al., 2007), suggesting that intracellular polyphosphate grains act as a reservoir of energy and phosphate. In fact, *M. ferroxydans* PV-1 has genes encoding polyphosphate conversion and stores it via ATP under the aerobic conditions of excess carbon and energy substrates. Although the pure culture of *M. ferroxydans* PV-1 cannot grow using any organic carbon, some single amplified genomes obtained from the Loihi Seamount implicate the metabolic potential for heterotrophy or mixotrophy of *Zetaproteobacteria* (Field et al., 2015). Taken together, we interpret that *Zeteproteobacteria* in the middle layer oxidize ferrous iron, produce stalks, and store polyphosphate grains inside the cell. In the top and bottom layers, they may be different metabolic and/or physiological state(s), i.e., gaining energy from iron-oxidation and/or heterotrophy without forming stalk-like structures. This might be an adaption mechanism of iron-oxidizing *Zetaproteobacteria*; however, mechanisms for their redox-sensing and energetic response to the environmental change still remain largely elusive.

In conclusion, we show that *Zetaproteobacteria* play significant ecological roles in iron cycling with other iron reducers and construct a unique microbial ecosystem in shallow hydrothermal iron-oxyhydroxide deposits. In addition, we first demonstrated that *Zetaproteobacteira* in the natural ecosystem store polyphosphate grains inside cells for energy storage and a phophate source with iron-oxidation and stalk formation. We also observed that *Zetaproteobacteria* without pholyphosphate grains with fewer stalk formations have largest population, indicating a possibility that *Zetaproteobacteria* changed their metabolism depending on the environments. These results provide some new insights into ecophysiology of iron-oxidizing *Zetaproteobacteria* and the microbial ecosystem in marine hydrothermal environments.

## Author contributions

TK, HO, and SK sampled and pretreated a core sample. TTH, YM and TMH performed the molecular works as DNA extraction and sequencing of 16S rRNA. TTH and YM carried out microscopic works including cell count and FISH. TTH analyzed sequencing data. TTH, SK, and FI designed of the work and drafted the manuscript, which was critically riveted by all authors. The final manuscript was approved by all authors.

### Conflict of interest statement

The authors declare that the research was conducted in the absence of any commercial or financial relationships that could be construed as a potential conflict of interest.
