# Supplementary figures and images for: Ecophysiology of Zetaproteobacteria Associated with Shallow Hydrothermal Iron-Oxyhydroxide Deposits in Nagahama Bay of Satsuma Iwo-Jima, Japan
Source: Front Microbiol. 2016 Jan 11;6:1554. doi: 10.3389/fmicb.2015.01554 (PMC4707226; doi:10.3389/fmicb.2015.01554)

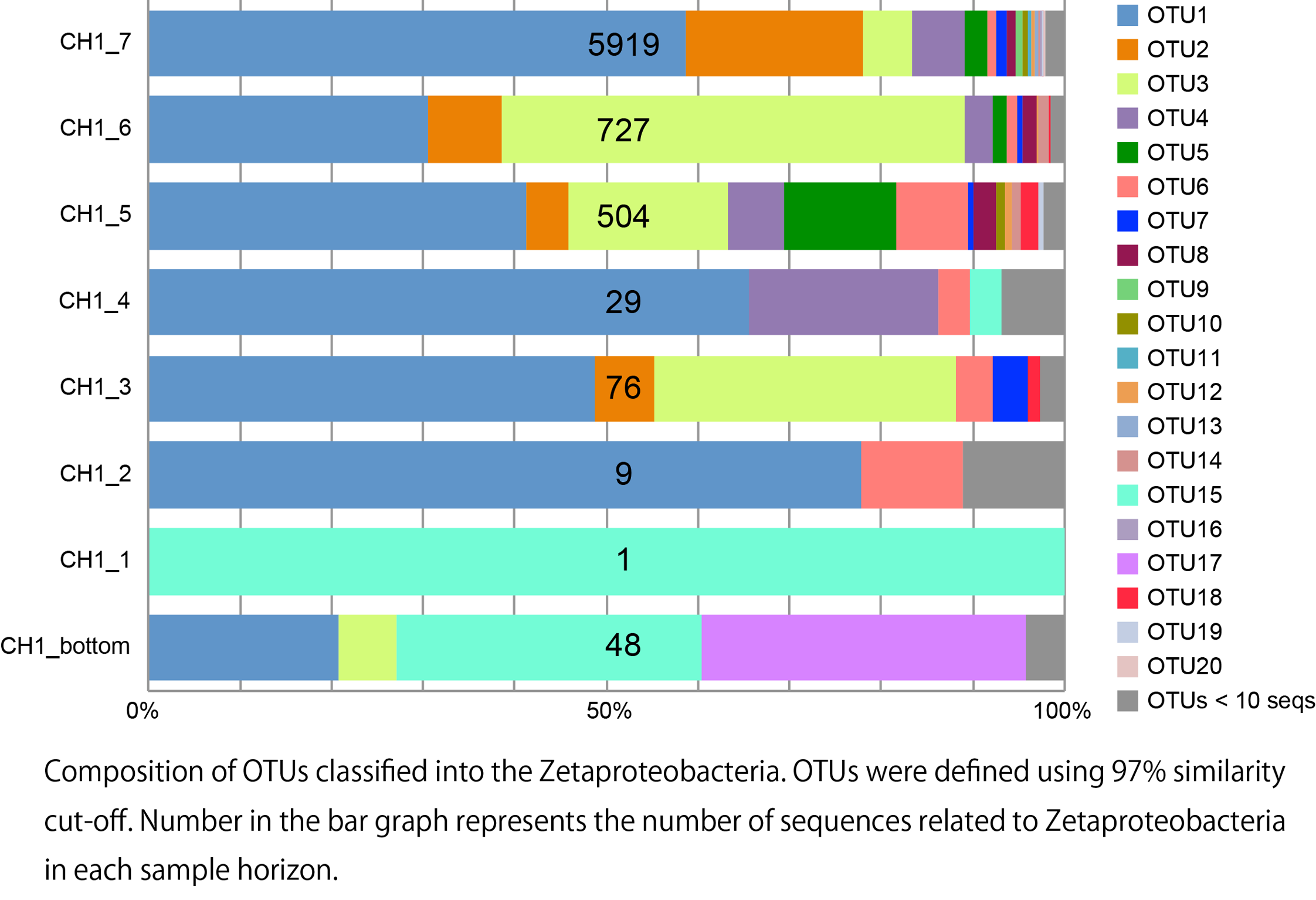

Supplement: Supplementary file 1 [file Image1.TIF]
